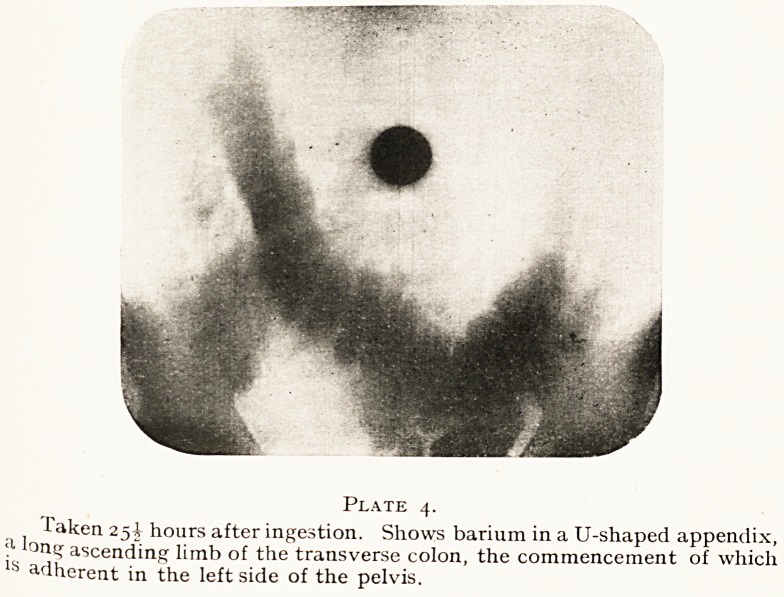# The Systematic Examination of the Abdomen

**Published:** 1919

**Authors:** R. G. P. Lansdown

**Affiliations:** Lecturer on Practical Surgery in University of Bristol; Surgeon to the Bristol General Hospital


### The Bristol
### Medico-Chirurgical Journal.

*" Scire est nescire, nisi id me*

*Scire alius sciret."*

SUMMER NUMBER, 1919.

### THE SYSTEMATIC EXAMINATION OF THE ABDOMEN.

The Presidential Address, delivered on December 11th, 1918, at the opening of the Forty-fifth Session of the Bristol Medico-Chirurgical Society.

BY

MAJOR R. G. P. LANSDOWN, R.A.M.C.T,,

*Lecturer on Practical Surgery in University of Bristol; Surgeon to the Bristol General Hospital.*

---

ALLOW me first of all to thank you for the honour you have done me in electing me to the Presidential Chair of your Bristol Medico-Chirurgical Society. It is a difficult thing for me to fill the chair with the distinction of many of my predecessors, but I have decided to address you on a subject which takes up much of my time and thought, one too which must perforce be of some interest to most members of our profession ; for every physician, surgeon and general practitioner is being consulted on the subject every day of his life, and upon his knowledge of the subject depends the future comfort and oftentimes the life of his patient.

I have taken as the title of my discourse to-night " The Systematic Examination of the Abdomen," or " How to Arrive at a Correct Diagnosis in Surgical Diseases of the Abdomen."

My method is to carry out an examination on the following lines: I first obtain as complete a history as possible of the present illness, then any points in the past and family histories which bear any possible relation to the present illness, noting of course the patient's age and occupation.

The patient is then stripped sufficiently for the surgeon to examine the whole abdomen without being hampered by having to pull articles of clothing up or down during the examination, the patient then lies on a couch or bed with a small pillow under the head. It is always wise to let the patient rest for a few moments after lying down, this short time is occupied in examining the tongue, noting any odour with the breath, and counting the pulse rate.

With a good light on the abdomen it is then carefully inspected, and the amount and extent of ordinary breathing movements observed, the presence of any tumour or abnormal swelling noted.

Note any other departure from the normal contour of the abdominal wall, such as prominence or depression in any region, whether the whole abdomen appears distended or scaphoid, the condition of the umbilicus and its shape. The presence of any unusual movements are noted; for example, the up and down movement of a tumour in the upper abdomen synchronous with respiration, visible peristalsis in one or more coils of the intestine or in the stomach, the exact site of origin and disappearance and the direction of the wave of contraction being noted.

If peristalsis is resulting from small intestine obstruction, it will be of the type spoken of as the ladder-rung variety, whereas if it is due to large intestine obstruction the peristalsis will be vertical in direction.

The presence of scars and their situation should be carefully noted. In cases of intestinal obstruction it is of great importance not to overlook scars in the skin, and to inquire as to their origin, as adhesions beneath the skin-scar or in the neighbourhood of the organs involved at a previous operation may be the cause of the trouble.

Again, it must never be forgotten in cases of intestinal obstruction how important it is to examine all points where hernia may be found.

Percussion is next performed, the area of liver dulness noted, whether it be normal, increased, diminished or absent. The fact that liver dulness is present does not of necessity exclude the diagnosis of perforation of the stomach or duodenum. Total disappearance of liver dulness in such a case must depend on (1) the length of time that has elapsed since the perforation took place, (2) on the size of the original opening, and (3) upon the nature of that which first tries to escape from the opening.

I was once asked by the late Professor J. Michell Clarke to operate upon a young woman whose stomach had perforated half an hour before he saw her and made the diagnosis; liver dulness was not diminished at all. At the operation, which was done within two hours of the occurrence of perforation, a piece of onion was blocking completely the orifice in the anterior wall of the stomach.

Crossing to the left of the middle line the splenic area is percussed; both renal and iliac regions and the hypogastric region should be examined in the same way, noting any evidence of unusual dulness, and if necessary the patient should be rolled over on to the side to ascertain the presence or absence of shifting dulness. It is seldom worth while to waste time by trying to map out the outline of the stomach by percussion; our ideas of its size and shape as mapped out by the combined methods of percussion and auscultation have been proved to be quite erroneous by the more accurate and scientific examination with the Röntgen Rays and the fluoroscope, after the ingestion of a bismuth or barium meal.

If there is any tumour in the epigastric region it should be noted whether it is dull or resonant on percussion.

Auscultation is of very little value in examining the abdomen. Occasionally it is useful in determining the presence of a patch of peritonitis or of locating the position of a stricture or narrowing of the bowel lumen.

The most important examination after inspection is that of palpation, and I do not think any of us live long enough to become as proficient in this form of examination as might be possible if we could continue with unflagging energy and intelligence beyond the time limit set by hospital authorities and the Almighty.

As in the examination of the female breast, so also in palpating the abdomen, it is possible to obtain the maximum of information by using the flat hand or the palmar aspects of the phalanges rather than the actual tips of the fingers. By use of the latter in any case suffering from an acute abdomen an unnecessary amount of pain is inflicted, and the examination is rendered more difficult therefore, as anything increasing pain will of necessity increase rigidity of the muscles. One of the most difficult things I know is to get the patient at times so to relax the abdominal wall as to allow of a thorough palpation being carried out ; in an acute abdomen with perforation of any portion of the intestinal tract this is quite impossible. Within a few moments of perforation taking place the abdominal wall becomes board-like and scaphoid in shape, later becoming distended by slow degrees. These symptoms are of so grave a nature that the surgeon should be asked to see the case immediately.

I always palpate the regions of the abdomen in order, commencing in the right hypochondrium, travelling across to the left, then coming back to the right side, examining in transit the left renal, umbilical and right renal regions, then from right to left again in the order right iliac, hypogastric and finally left iliac.

By this systematic method of palpation one is able to decide whether any particular region is more resistant than others, or whether guarding contraction of the muscles follows on examination in any one or more regions. The hand next grasps the loins in turn, the right hand is placed with the fingers over the right side of the abdominal wall and the thumb in the interval between the last rib and the crest of the ilium. When the patient takes a deep inspiration and forces the diaphragm down the hand may grasp a tumour, or a misplaced or enlarged viscus or portion of a viscus. Passing round to the other side of the bed or couch, the same examination is carried out on the left side, the left hand being used in exactly the same manner as was the right hand on the right side. Where it is not possible easily to pass to the other side of the bed, the left side can be examined by placing the right thumb over the left abdominal wall and the fingers in the loin.

In connection with examination by palpation, I must refer to the presence of hyperalgesia in abdominal disease, and say a few words of its importance in helping us to arrive at a correct diagnosis. An article appeared in *The Practitioner* of August, 1916, by David Ligat, giving the result of his investigations, conducted on the lines laid down by Sir James Mackenzie in *Symptoms and their Interpretation*. He considers that " all the pain that a patient complains of, and the tenderness that an observer can elicit, would seem to be due to a true *viscero-sensory reflex*, and not in any way to pain or tenderness felt in the organ itself. Various methods of eliciting reflex responses were tried by Ligat ; that which he found to yield definite and demonstrative results he describes as follows : " Grasp the skin and subcutaneous tissue firmly between finger and thumb, and draw them away from the deeper layers of the abdominal wall. If an hyperalgesic area be present the patient winces, and one can tell by the patient's expression when such an area is being stimulated."

After long and careful observation Ligat was forced to the conclusion that these points corresponded anatomically with the subcutaneous distribution of certain branches of particular nerves. The amount of pain in different cases varies very considerably. Where there is general evidence of acute inflammation of a viscus the pain produced by pinching up the skin may be elicited over a large area of skin, corresponding perhaps to two or even three spinal segments, but by carefully mapping out the point of maximum hyperalgesia the actual organ affected can generally be decided upon. In other cases where there is no evidence of acute inflammation and vague abdominal pain is the chief complaint, very careful examination is necessary, closely watching the alteration produced in the patient's facial expression when the skin is pinched up. It is in this type of case that I have myself found the greatest amount of assistance from this method of examination. The sensation produced is not always described by the patient as one of actual pain, it is frequently described as "pricking," "curious," "different from a similar pinch on the other side." This diagram copied from Ligat's paper gives a good idea of the different areas affected in different viscera. In all cases where I have to examine an abdomen I invariably test these areas for hyperalgesia.

There are three so-called "signs" recognised in connection with abdominal palpation associated with the name of those who first pointed out their value. They are :—

1. *The McBurney Sign.*—I quote from Kelly's monograph on *The Vermiform Appendix and its Diseases.* "As to the location of the pain as a significant factor in making a

PLATE 1.—*Diagrammatic illustration of the hyperalgesic points.*

1. Hyperalgesic area when present in gall-bladder lesions. 2. In appendix lesions. 3. In right Fallopian tube lesions. 4. In gastric and duodenal ulcers. 5. In small bowel lesions. 6. In large bowel lesions. 7. In left Fallopian tube lesions.

PLATE 2.

Taken $4\frac{1}{2}$ hours after ingestion of barium meal. Shows most of the meal in the small intestine, quite low down in the pelvis, and the head of the column in the ascending colon. There is a faint indication of the last few inches of ileum below and to the right of the sacro-iliac synchondrosis.

diagnosis of 'appendicitis,' the value of the 'McBurney point' is now so universally known that it is most interesting to read the first statements of the author relative to the matter. 'The exact locality of the greatest sensitiveness had seemed to me,' he says, 'to be usually one of importance. Whatever may be the position of the healthy appendices found in the dead house—and I am aware that its position when inflamed varies greatly—I have found in all my operations that it lay, either thickened, shortened or adherent, very close to its point of attachment to the cæcum. This, of course, must in early stages of the disease determine the seat of greatest pain on pressure. And I believe that in every case the seat of greatest pain, determined by the pressure of one finger, has been very exactly between an inch and a half and two inches from the anterior spinous process of the ilium on a straight line drawn from that process to the umbilicus. This may appear to be an affectation of accuracy, but so far as my experience goes the observation is correct.'"

2. *Murphy's Sign.*—Murphy wrote (in the *Medical News*, vol. i., 1903, p. 825): "The most characteristic and constant sign of gall-bladder hypersensitiveness is the inability of the patient to take a full inspiration when the physician's fingers are hooked up deep beneath the right costal arch below the hepatic margin. The diaphragm forces the liver down until the sensitive gall-bladder reaches the examining fingers, when the inspiration suddenly ceases as if it had been shut off. I have never found this sign absent in a case of calculus or in infectious cases of gall-bladder or duct disease." This last statement I can repeat as the result of observation in all cases of this kind that I have examined since I read his paper in 1903.

3. *Boas's Sign.*—Boas writes (*Munch. med. Woch.*, April 15th, 1903, p. 604): "Least recognised as a symptom of cholelithiasis is tenderness over the posterior surface of the liver. When well marked it extends laterally from about an inch external to the spines of the vertebræ to the posterior axillary line, and vertically from the eleventh dorsal to the first lumbar spines. To demonstrate it the finger should be pressed against a point to the right side of the tenth dorsal spine; then against successive points in lines running horizontally outwards, opposite the other spinous processes, down to the first lumbar spine, first on one side, then on the other. This symptom, if present during the acute attack, is also invariably present in the intervals; that is, if once present it is always present, and is therefore of special diagnostic value in the later stages. Occasionally it may be found years after the last attack of colic. Conversely, if absent in the acute attack, it is not found in the intervals. It is usually sufficient to map out the areas of tenderness with the finger, but when there is a doubt as to whether the right side is the more tender, greater accuracy may be obtained with the faradic or galvanic current. When, as often occurs, the lower edges of the liver and the gall-bladder are not tender, the discovery of the second or third areas of tenderness may, in conjunction with other symptoms, often decide the diagnosis. The presence of one or more of these areas indicates also that though no attack of colic may have occurred for some time the patient still requires supervision and treatment."

Commenting on this sign, Sir Berkeley Moynihan says (*Gall-stones and their Surgical Treatment,* page 117): "I consider the search for the tender area a necessary part of the examination of all patients who suffer from gall-stone diseases, or in whom the existence of this disease is suspected. It is undoubtedly a sign of great value." I have myself used this sign in all cases of suspected gall-stone disease that I have examined since the end of 1904, and have only found gall-stones present in two cases where I had not proved the presence of the tender areas. In association with the presence of hyperalgesia in the gall-bladder area, this sign is of the greatest possible value in helping us to arrive at a correct diagnosis in those cases where a difficulty exists in determining whether we are dealing with appendicitis or gall-stones, or a tumour in the right side of the abdomen. I have not found this sign present in a fair number of cases of malignant disease of the gall-bladder in which gall-stones were not demonstrated at operation.

I have noticed that in cases of adhesions of the gall-bladder involving the pylorus or the duodenum, and in cases of pyloric ulcer whether on the duodenal or gastric side of the pylorus, an area of tenderness similar to that obtained in Boas's sign is to be elicited over a corresponding area between the seventh and tenth thoracic spines vertically. This corresponds to the area of distribution of the cutaneous branches of the posterior divisions of the sixth, seventh and eighth thoracic spinal nerves. The area over which the sign described by Boas is obtained corresponds to the distribution of the cutaneous branches of the posterior divisions of the ninth and tenth nerves. If investigation is further pursued on these lines, we shall possibly find that there is a definite area of hyperalgesia obtained for diseases of the larynx, pharynx and œsophagus.

I should like here briefly to recite the notes of four cases which illustrate what I have just said about hyperalgesia, Boas's and Murphy's signs. They are as follows :—

**Case 1.**—Mrs. M., æt. 35, wife of a doctor, seen July 8th, 1916, at 8.30 p.m. Taken ill suddenly at 11.30 last night, with severe pain in the back ; 6 a.m., vomiting and repeated vomiting until 9 a.m. Has had two or three similar but less severe attacks every year for the last three or four years. Her father died at age of 74 of carcinoma of the liver, her mother at the age of 54 of cancer of the pancreas. In the right renal region is a sausageshaped tumour, quite hard and nodular, which passes up and disappears into the hepatic region. It comes down on deep inspiration. There is no area of resonance between the tumour and the liver edge. Gall-bladder hyperalgesia, Murphy's and Boas's signs are all present and very well marked. Eleven days later the gall-bladder was opened and drained, a large number of stones having been evacuated. The swelling had previously been diagnosed as a cancerous tumour of the ascending colon, on account of the hardness and of the situation.

**Case 2.**—Mrs. S., æt. 52, seen September 15th, 1916. There was a history of gall-stone colic followed by jaundice eight years ago and again last Christmas. Hyperalgesia was absent, but Boas's and Murphy's signs were both present and well marked. In the right hypochondrium, one inch to the right of the middle line, and half-way between the umbilicus and the ensiform cartilage, deep pressure always produced marked wincing; it is interesting to note that tenderness in that spot is frequently found in association with duodenal ulcer. She was operated on September 24th. Between the time of my first seeing her and of the operation she had passed two gall-stones. The gall-bladder was found adherent to the anterior abdominal wall, the transverse colon and the second portion of the duodenum. A fistula was existing between the gall-bladder and the duodenum, this accounted for the point of tenderness mentioned above. The gall-bladder and cystic duct were removed with stones *in situ*, the stones in the bladder and cystic duct were white and pearl-like in appearance.

**Case 3.**—Mrs. W. W. P., æt. 61, seen November 30th, 1916. Seven years ago had an attack of indigestion with pain in the upper abdomen, has had numerous attacks of niggling pain since. Some months ago found a difficulty in sitting forward, as something hard got in the way. A tumour was found by the medical attendant in the right side of the abdomen, which was not connected with the pelvis, and was thought to be a malignant growth in connection with the ascending colon. The bowel for the last thirty years has been relieved twice daily, and the motion has always been of a fluid consistency; there have never been any blood or mucus in the stools, and she has never been constipated. There is a large sausage-shaped tumour coming down from under the lower border of the right lobe of the liver, which comes down farther on taking a deep inspiration. There is no resonance between the tumour and the liver edge, no impulse into the loin, and no dilatation of the cæcum. No Boas's or Murphy's sign and no hyperalgesia present. A diagnosis of impaction of a gall-stone in the cystic duct with hydrocele of the gall-bladder was made. The abdomen was opened on December 3rd, when a large distended gall-bladder partially covered by a Riedel's lobe was delivered, on raising which a stone was seen impacted in the cystic duct. The cystic duct was clamped and ligatured, and was removed with the gall-bladder *in situ*.

**Case 4.**—Mrs. C., æt. 56, seen January 20th, 1917. For some considerable time past has suffered from heart attacks; they had ceased for a time, but returned three weeks ago. She has had no vomiting, no diarrhœa, and no constipation. There is a tumour in the right hypochondrium extending from the rib margin to a point one inch below and immediately to the right of the umbilicus, the lower end of the tumour is stony hard and rounded. The edge of the liver can be felt on each side of the tumour, and is much lower than normal; indeed, that part of the right lobe which is external to the tumour almost reaches the iliac crest. There is no area of resonance between the tumour and the liver; there is no impulse into the loin and no dilatation of the cæcum. Boas's and Murphy's signs are both absent, but gall-bladder hyperalgesia is well marked. The diagnosis made was occlusion of the cystic duct by a stone, with probably a large stone at the distal end of a much distended gall-bladder. At the operation performed by the medical attendant on January 21st the condition found was exactly as described.

In almost all cases of abdominal disease it is a wise rule to examine the rectum as a routine procedure; in cases where the patient complains of suffering from piles or from hemorrhage from the anus, or of frequent diarrhœa or constipation or of the passage of mucus with the fæces, it is in my opinion criminal to neglect to carry out such an examination.

The presence or absence of a tumour in the pelvis should always be noted. I vividly recall one case some few years ago of a woman who was suffering from extremely obstinate constipation. I was asked to see the case with a view to remove the colon, another surgeon having stated that it was eminently a suitable case for that method of surgical treatment. I was supplied with a copy of very elaborate notes, but on going through them very carefully before examining the patient was struck with the fact that no mention was made of any rectal examination having been made. I made a careful examination of the abdomen and then of the rectum ; the latter was absolutely blocked by a large, tender swelling in Douglas's pouch, which was diagnosed as, and at a subsequent operation proved to be, an adherent and inflamed ovarian cyst, removal of which absolutely cured the constipation.

Other methods of examination are also necessary in arriving at a correct diagnosis. We have before us usually an analysis of the urine in any case for investigation, whether abdominal or not. A blood count is sometimes helpful, as in distinguishing between Banti's disease and splenic leukæmia. It is useful sometimes also in cases where it has been difficult to decide whether suppuration has occurred, though it must never be forgotten that leucocytosis is by no means pathognomonic of suppuration.

Lastly, we have the more modern method of examination of the intestinal tract by means of a fluoroscope after the ingestion of a meal which is opaque to the passage of Röntgen Rays. This method of investigation is of the greatest possible value in coming to a correct diagnosis in chronic abdominal conditions ; but it is readily seen that it can be of very little assistance, if any, in that group of abdominal conditions which is now so generally spoken of as the acute abdomen.

If the fluoroscope is used in arriving at a correct diagnosis in abdominal disease, it is essential that the surgeon called upon to make the diagnosis should himself be present at the examination, and not expect the skiagraphist to supply him with three or four plates showing a bismuth meal at certain regions of the intestinal tract taken at certain times ; looked at *de novo* such plates can convey very little, if any, information to any man who was not present at the examination.

When the patient is on the X-ray table, and the surgeon is able to see the shadow cast by the meal, and at the same time by digital manipulation to separate different coils of intestine, or note the speed with which the meal is discharged from the stomach or reaches definite sites in the intestinal tract, or observe the absence or excess of peristalsis, he gains a large amount of knowledge which he would have lost had he relied upon the plates supplied. Provided the surgeon has been present at the examination, the plates of conditions he has actually seen are of the greatest assistance in refreshing his memory when proceeding to operate on the case or to write a report thereon. It is generally wise to see the patient in the erect as well as in the recumbent position, as the position of the different viscera vary both in health and disease according to the position of the trunk.

The following case illustrates well the difficulty of arriving at a correct diagnosis from skiagrams alone without personal examination of the abdomen with the fluoroscope or without making a thorough abdominal examination on the lines laid down in this paper.

T.P., male, æt. 30, was admitted on September 11th, 1918, with the diagnosis of gastro-enteritis, which was later changed to intestinal adhesions. On September 20th I was shown some plates (of which I am going to show the lantern slides) which had been taken by the skiagraphist, and which were accompanied by a report which stated that the "findings point to adhesions (?) at hepatic flexure." I asked to be allowed to examine the patient before being expected to express an opinion as to his diagnosis. I saw him on September 23rd. For ten years past he has suffered from severe attacks of abdominal pain, and for the past three years from attacks of vomiting and diarrhœa. The cæcum was dilated, tenderness was well marked at McBurney's point, appendicular hyperalgesia was present, and the rectum was loaded. I wrote down my deductions from the examination of the plates and the abdomen as follows :—

1. Adherent appendix, kinked.
2. Fixed caput cæci.

3. Jackson's veil round cæcum and ascending colon.

4. ? Ascending colon and first limb of transcending colon adherent together.

5. Centre of transcending colon adherent in left side of pelvis.

On September 24th he was operated on and the following findings noted :—

1. Adherent kinked appendix, removed.

2. This released a fixed caput cæci.

3. Large Jackson's veil all round cæcum and ascending colon.

4. At hepatic flexure the veil causes adhesion between upper two or three inches of ascending colon and first limb of transcending colon.

5. Centre of transcending colon fixed in left iliac fossa by overlying coils of small intestine.

The giants of medicine in the past who claimed Guy's as their Alma Mater, Bright, Addison, Wilkes, Moxon, and Mahomed, attained the high standard of their diagnostic acumen by the regularity with which they attended the *post-mortem* room, and examined the pathological conditions present to account for the symptoms which they had observed during their patients' last illness. These giants were famed for their knowledge of diseases of the kidney, blood, heart, lungs, and liver. There are and have been very few giants among the physicians who have obtained their reputation by their skill in abdominal diagnosis. The reason for this is not far to seek. The physicians have not the opportunity of looking into their patients' abdomens to the same extent that the surgeons have, and it is noted with regret that so many of them do not take the opportunities offered to them by their surgical colleagues of being present to see their cases on the operating table. Far more is learnt by seeing a series of abdominal operations on patients whom you have seen and

PLATE 3.

Taken $8\frac{1}{2}$ hours after ingestion. Shows barium in the appendix, the ascending colon adherent to the first limb of the transverse colon, and the head of the column half-way through the transverse colon.

PLATE 4.

Taken $25\frac{1}{2}$ hours after ingestion. Shows barium in a U-shaped appendix, a long ascending limb of the transverse colon, the commencement of which is adherent in the left side of the pelvis.

examined clinically beforehand than by attending any number of *post-mortem* examinations, for by far the greatest number of abdominal cases operated on recover, and do not reach the *post-mortem* room on account of the conditions for which the operation was performed, and secondly, those that do die have been investigated on the operating table, and there is usually no necessity for a further examination after death.

In this connection the two following quotations from C. L. Greene of St. Paul (*Medical Diagnosis*, page 1) are worth remembering :—

1. "Accurate diagnosis is prerequisite to accurate prognosis and effective treatment."

2. "Diagnosis demands a sufficiency of facts, truthfully recorded, intelligently sifted, and viewed without bias or prejudgment."

The safety and future welfare of the patient are enormously enhanced if the doctor can make a correct diagnosis of the abdominal condition which is causing the symptoms at a sufficiently early date, and is willing to call in a surgeon as soon as the diagnosis is made. Many a useful life has been lost by the practitioner waiting until the diagnosis is all too evident, and the valuable moment when a successful operation might have been performed with little or no risk has been allowed to pass.

## Figures and Tables

**Plate 1. f1:**
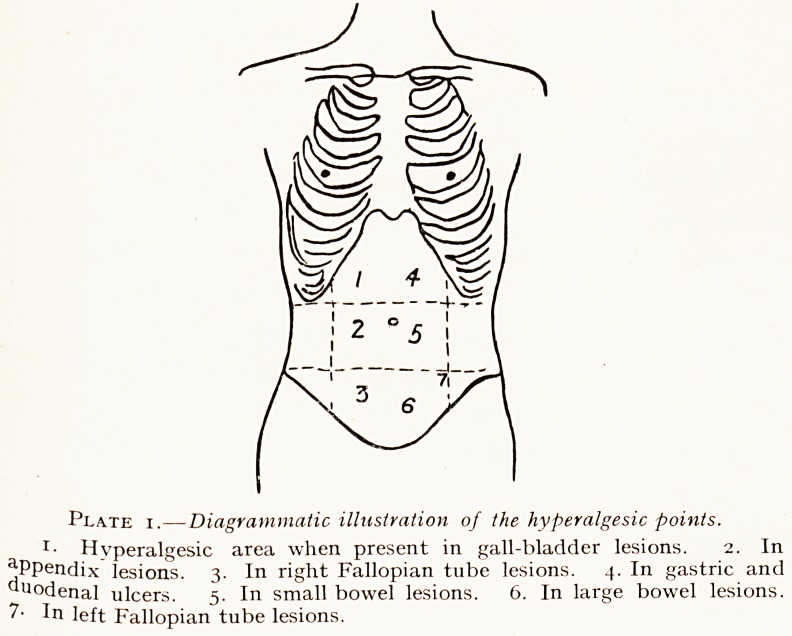


**Plate 2. f2:**
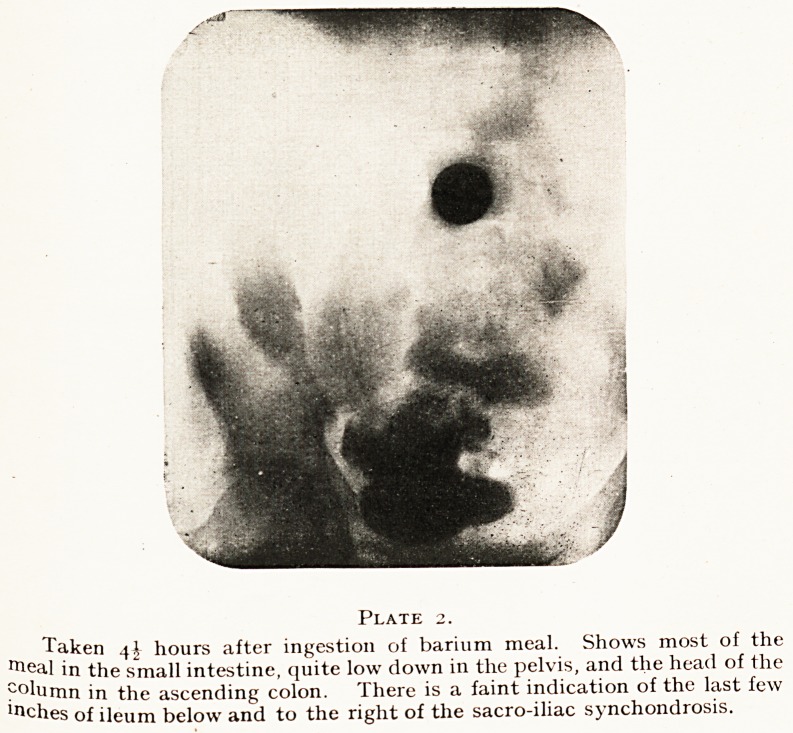


**Plate 3. f3:**
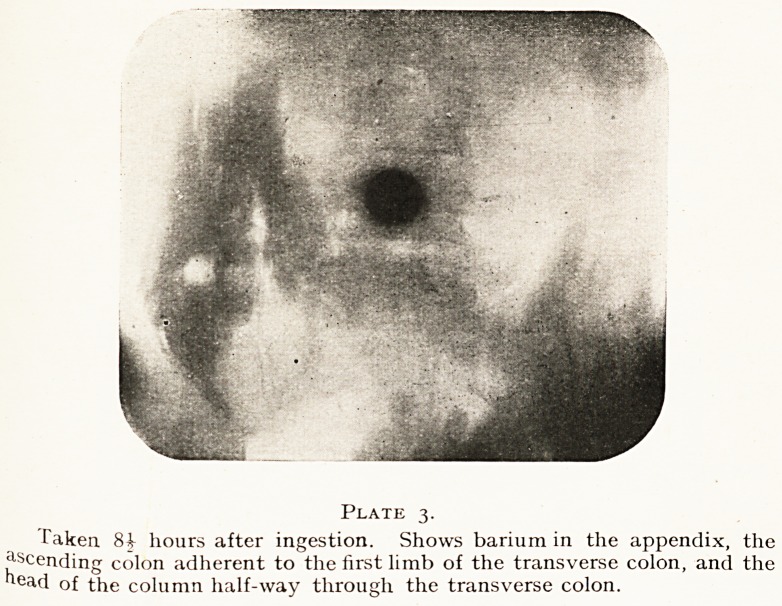


**Plate 4. f4:**